# A health facility based case-control study on determinants of low birth weight in Dassie town, Northeast Ethiopia: the role of nutritional factors

**DOI:** 10.1186/s12937-018-0409-z

**Published:** 2018-11-06

**Authors:** Semira Ahmed, Kalkidan Hassen, Tolassa Wakayo

**Affiliations:** 1 Gambella Regional State Health Bureau, Gambella, Ethiopia; 2 Jimma University, Faculty of Public Health, Jimma, Ethiopia

**Keywords:** Low birth weight, Maternal nutritional status, Determinants, Ethiopia

## Abstract

**Background:**

Low birth weight remains a major public health problem affecting developing countries. Evidence shows that low birth weight has long lasting negative health consequences through its contribution to stunting, mental impairment and non-communicable chronic diseases in later life. Thus, it is worth investigating the role of nutritional factors as determinants of low birth weight to suggest nutritional interventions to curb its negative health outcomes. This study aimed to investigate the determinants of low birth weight with main focus on the role of nutritional factors in Ethiopia.

**Methods:**

A facility-based case-control study was conducted from 3 February to 29 April, 2017. The data were collected using structured, pretested interviewer-administered questionnaire in all public health facilities of Dessie Town. Anthropometric measurements were made following standard procedures for both mothers and their newborns. Consecutive live births of < 2500 g and two succeeding normal weight babies were selected as cases and controls, respectively. Data were entered in to Epi-data software version 3.1, and exported to SPSS version 21, and analyzed using frequency, mean and percentage. Factors with *p* < 0.25 during bivariate analyses were entered into a multivariable logistic regression model to determine significant determinants of LBW. Statistical significance was considered at *p* < 0.05. Results were reported with odds ratio and 95% CI.

**Results:**

Mean ± SD of birth weight (g) was 2138 ± 207 for cases and 3145 ± 415 for controls. After adjusting for potential confounders using multivariable logistic regression analysis, the absence of iron and folate supplementation, receiving no nutritional counseling and consuming no additional meal, maternal undernutrition, maternal anemia and inadequate dietary diversity during the current pregnancy were found to be significant determinants of low birth weight in our study.

**Conclusion:**

Lack of nutritional counseling, absence of additional meal intake and iron and folate supplementation during pregnancy, and maternal undernutrition, maternal anemia and inadequate maternal dietary diversity were significant determinants of low birth weight. The importance of nutritional counseling, improving iron and folate supplementation during pregnancy, and nutritional status of pregnant women need to be strengthened to reduce the incidence of LBW in Ethiopia. In addition, behavioral change communications targeting pregnant women to improve women dietary diversity and their extra meal intake practice need to be enhanced in Ethiopia.

## Background

Low birth weight (LBW) is defined by the World Health Organization (WHO) as weight at birth less than 2500 g. Low birth weight is more common in developing countries and contributes to a range of poor health outcomes in later life [1].

Most of the LBW infants are born either preterm or have intrauterine growth retardation (IUGR). While most of the LBW newborns in developed countries are preterm, these are full-term infants who are small for gestational age (SGA) in developing nations. The causes of SGA include poor maternal nutritional status at conception, low gestational weight gain due to inadequate dietary intake or due to excess expenditure of calories (hard work), and short maternal stature resulting from early childhood undernutrition. Infections, malaria, anemia, acute and chronic infections that could result in maternal undernutrition can lead to SGA baby and subsequent LBW infant [2–4]. The incidence of LBW is estimated to be 15% to 20% worldwide, representing more than 20 million births a year [5]. The great majority of low birth weight occurs in low- and middle-income countries and especially in the most vulnerable populations accounting for 28% in south Asia, 13% in sub-Saharan Africa and 9% in Latin America [6]. Among children born with a reported birth weight in Ethiopia, 13% were LBW babies [7].

LBW has many negative health consequences for the infants. It contributes to 60% to 80% of all neonatal mortality and morbidity [3, 8]. LBW infants are many times more likely to end up with long-term handicapping conditions [8]. They are also at higher risk of infection, poor mental development, stunting and developing non-communicable chronic diseases in later life [2, 3, 9–11]. These consequences are more pronounced when LBW coincides with poor maternal nutritional status thereby adversely affecting the optimum fetal and child nutrition and development, cognitive function, motor and socio-emotional development and economic productivity being adult [12, 13]. Contrary to its known negative health consequences, studies have identified common prevalence and incidence of LBW in many developing countries; possible risk factors have also been reported. In India maternal age (< 19 years), rural residence, maternal weight (< 45 kg), gestational age (< 37 week), bad obstetric history and pregnancy induced hypertension had a strong association with LBW [14]. Low socio-economic status, anemia, primiparity, short maternal height and less than average weight had also strong association with LBW in Pakistan [15]. Other studies in developing countries have shown correlations of maternal nutritional status, young maternal age, bad maternal obstetric history, maternal anemia, rural residence of mothers, absence of maternal antennal care (ANC) follow up, prematurity, and birth interval with occurrence of LBW [13, 16–21]

In Ethiopia, maternal and environmental factors [22], inadequate ANC follow-up, unwanted pregnancy, sex of the neonate and pregnancy type [23], lower socioeconomic status, not attending ANC, maternal undernutrition, and experience of physical violence during the index pregnancy and longer time to walk to health facility [22, 24], urban residence, prematurity, having weight loss and receiving no additional diet during pregnancy, and multiple gestations [25, 26], marital status, monthly family income, and maternal HIV status were predictors of LBW babies [26]. The most recent study in northern Ethiopia also reported some non-nutritional factors predicting LBW [27].

Though there are some studies undertaken in Ethiopia aiming at determining predictors of LBW [22, 23, 25, 28–34], these studies considered only environmental, socioeconomic, demographic, maternal, and newborns characteristics and the majority of these studies used cross-sectional design. Few studies [24, 35] have been conducted with regard to maternal nutritional status and dietary practices towards contributing to the incidence of LBW in Ethiopia. However, these studies overlooked some important nutritional factors that may contribute to LBW babies in Ethiopia. Therefore, the aim of this study was to investigate nutritional determinants of LBW among newborns delivered at public health facilities in Dessie Town, Northeast Ethiopia.

## Method and material

### Study design and subjects

A facility based unmatched case-control study was conducted from 3 February to 29 April 2017 in all public health facilities in Dessie Town, Northeast Ethiopia. Dessie Town, located in Amhara regional state of Ethiopia, is located at 401 km from Addis Ababa, the capital of Ethiopia and 523 km from Bahir Dar, the capital of the region. The town is situated at a latitude and longitude of 11°8′N 39°38′E, with an elevation between 2470 and 2550 m above sea level. It has ten sub-cities and six rural kebeles. According to the 2007 National Censes, the total population of the town was about 151,174; of whom 72,932 were men and 78,242 were women; 79.44% were urban inhabitants living in the town, the rest of the population was living in rural kebeles surrounding the town. All neonates delivered in all public health facilities of Dessie Town were considered the source population of the study. Cases were those neonates delivered with birth weight of < 2500 g while controls were those neonates delivered with birth weight of ≥2500 g in each health facility of the town.

### Sample size determination

The sample size was determined using a proportional difference approach for case-control study using Epi-Info statistical software package (Version-7) taking into account maternal anemia as main exposure variable and considering 95% confidence level, 80% power of the study and control to case ratio of 2:1 for estimating low birth weight. As such, we considered that the percent of controls exposed (having anemic mothers) among the controls was 11.6% [36]. Assuming a 15% difference (increase) in cases that was assumed in advance, proportion of cases with exposure became 26.6% producing the least extreme Odds Ratio of 2.76 to be detected. Accordingly, after adding 10% non-response rate to each, 95 cases and 191 controls were estimated making a total sample size 286 subjects to be included in the study.

### Sampling procedure

All of ten public health facilities in the Dessie Town (Two hospitals: Dessie Referral Hospital, and Borumeda Hospital, and eight health centers: Bwanbwawua, Dessie, Segno Gebya, Gerado, Tita, Kurkur and Boruslasie health centres) were included in this study to compensate for short data collection period to acquire the required number of cases. The allocation of the required number of study subjects to each health facility was based on the proportion of number of deliveries during same time interval of the preceding year (from records) in each health facility. After allocating the total sample size to each health facility using population proportional to size approach, consecutive live births (< 2500 g) each health facility were selected as cases and two babies with normal birth weight (≥2500 g) succeeding each case were selected as controls. Neonates with major congenital anomalies such as macrocephaly, neural tube defect and limb reduction, whose mothers are known diabetic, and those whose mothers didn’t know their last menstrual period (LMP) were excluded from the study while cases and controls with term singleton live-births were included in the study. Few deaths were reported with in 1 h of delivery and they were also included in the study.

### Ethical consideration

Ethical clearance letter was obtained from Jimma University Institute of Health, Research and Community Service Office. Then, written letter of support to conduct the research was obtained from Ethical Review Board of Dessie Town Health Administration Department and presented to respective public health facilities in the town. The interviewers discuss the issue of confidentiality and obtained verbal consent before the actual interview was administered.

### Data collection

Data were collected by trained nurses using face to face interview and structured and pretested questionnaire was used to collect data. The interviews were conducted at each health facility after the mother had given birth and the neonate weight was measured within 1 h of delivery. Ten data collectors, six midwives and four nurses were recruited to accommodate the wide range of health facilities. Four first degree nurses supervised data collection in all health facilities while the principal investigator had overall role of facilitating research works during data collection.

### Anthropometric measurement

Anthropometric measurements were measured following standardized techniques. Weights of the newborns were taken within 1 h after delivery using a balanced digital Seca scale (Germany). The scales were always calibrated using a material with standard weight and the reading on each scale was taken to zero level before weighing each newborn. Height of the mother was measured using height board while the mother was in standing position. Each height was taken to the nearest 0.1 cm. Mothers were asked to stand without shoes in front of the height board, with the head erect and the arms hanging naturally at the sides. The mid-upper arm circumference (MUAC) of the mother was measured after delivery using flexible non-stretchable standard tape measure. The circumference was measured at the mid-point between the tip of the acromion process of the scapula and olecranon process of the ulna. Measurements were taken while the right arm was hanging relaxed, to the nearest 0.1 cm.

### Dietary assessment

The minimum dietary diversity for women (MDD-W) was collected using 24-h recall method by using MDD-W tool [37]. Briefly, the pregnant women were asked to recall the foods they had consumed in the previous 24 h, first spontaneously followed by probes to ascertain that no meal or snack was left out. A detailed list of all the ingredients of the dishes, snacks, or other foods consumed was generated to enable better classification of mixed dishes. The foods were then categorized into 10 food groups. Then, tabulation of the MDD-W was done using SPSS and a pregnant woman was assigned in the adequate dietary diversity group if her MDD-W ≥ 5 food groups or in the inadequate group if her MDD-W < 5 food groups.

### Micronutrient intake and hemoglobin level

Iron and folic acid (IFA) supplementation along with history of ANC (how many times did the mother visit health facility during pregnancy to receive routine ANC services) were asked for each mother. The hemoglobin level of each mother was taken from her health card as hemoglobin is routinely done for each mother receiving delivery services in each public health facility. WHO recommends four ANC visits during pregnancy and visits less than four were considered inadequate.

### Data quality control

Data were collected by trained data collectors after full understanding the objectives of the study, contents of the questionnaire and method of data collection. The questionnaire was prepared in English and translated to Amharic and back translated to English to keep the consistency of the questions and increase understanding with respondents. Both supervisors and principal investigator were closely following the data collection process. The questionnaire was pretested on 5% of the sample size in another health facility out of the study area before the actual data collection was implemented. The collected data were checked for completeness and consistency on daily basis by principal investigator and supervisors.

### Wealth index

Socioeconomic index was developed as follows: first all study participants were asked about the ownership of fixed assets by their household with a score 1 given to those who own the asset and score of “0” given to those who did not own. Then all items asked were assessed for internal consistency and showed to be reliable with a cronbach’s alpha value of 0.82 (> 0.7 is considered as reliable). Then principal component analysis was used to develop the wealth index. The first factors were taken and rank ordered into tertiles.

### Statistical analysis

Data were checked for completeness and consistencies, and then edited, coded and entered using Epi-data version 3.1, and then exported to SPSS version 21 for further analysis. Variables were checked for missing values before analysis. Descriptive statistics were computed for all variables according to their type. Means, medians and standard deviations were computed for continuous variables. The categorical variables were described by their frequencies. Normality of continues variables was checked using the Kolmogorov–Smirnov test. Bivariate analyses were done to identify the candidate variables for multivariable logistic regression model to determine risk factors of LBW. Accordingly, any variable having *p* ≤ 0.25 was considered as a candidate variable for multivariable analysis and entered into multivariable logistic regression model using backward elimination stepwise likelihood ratio method. All tests were two-sided and *p* < 0.05 was considered statistically significant. The results were reported as Odds Ratio (OR) and 95% Confidence Interval (CI).

## Results

### Socio demographic characteristics

A total of 279 mothers with their respective newborns (93 cases and 186 controls) were included in the study making the response rate of 97.6%. The mean ± SD of birth weight was 2138 ± 206 g for cases and 3145 ± 415 g for controls. A higher proportion of newborns were males both in cases (52.7%) and controls (62.9%). The mean ± SD of maternal age among the cases was 27.3 ± 5.5 years and it was 26.2 ± 4.7 years among controls. The majority, 82.8% vs. 79.6%, of mothers among the cases and the controls were in the age group of 21–35 years, respectively. The largest proportion, 82.8% vs. 90.3%, of mothers among cases and controls were Amhara, while 74.2% of mothers among cases and 75.3% among controls were Muslim in religion. Among the mothers of the cases and the controls, 34.4% and 17.2% respectively, had informal education. Overall, most of the mothers in both cases and control groups were married (93.5% and 92.5%, respectively). Fifty-eight (62.4%) of the mothers among the cases and 54.3% of the mothers among controls were in lower wealth index category. Moreover, 44.1% of mothers among cases and 40.3% among controls were living in rural setting. The higher number of mothers in both cases (69.9%) and controls (48.9%) were housewives followed by employed (18.3% and 33.9%, respectively) (Table 1).

**Table 1:** Distribution of socio-economic and demographic characteristics among mothers of cases and controls in public health facilities of Dessie Town, Northeast Ethiopia, 2017

Variables	Cases	Controls	Total
	N (%)	N (%)	N (%)
Infant sex			
Male	49 (52.7)	117 (62.9)	166 (59.5)
Female	44 (47.3)	69 (37.1)	113 (40.5)
Maternal age (year)			
≤ 20	9 (9.7)	32 (17.2)	41 (14.7)
21–35	77 (82.8)	147 (79.6)	225 (80.6)
> 35	7 (7.5)	6 (3.2)	13 (4.7)
Residence			
Rural	41 (44.1)	75 (40.3)	116 (41.6)
Urban	52 (55.9)	111 (59.7)	163 (58.4)
Religion			
Muslim	69 (74.2)	140 (75.3)	209 (74.9)
Orthodox	20 (21.5)	42 (22.6)	62 (22.2)
Others	4 (4.3)	4 (2.2)	8 (2.9)
Ethnicity			
Amhara	77 (82.8)	168 (90.3)	245 (87.8)
Oromo	10 (10.8)	7 (3.8)	17 (6.1)
Gurage or Tigrae	6 (6.5)	11 (5.9)	17 (6.1)
Marital status			
Married	87 (93.5)	172 (92.5)	259 (92.8)
single, separated or divorced	6 (6.5)	14 (7.5)	20 (7.2)
Maternal Educational			
Informal education	32 (34.4)	32 (17.2)	64 (23.0)
Formal education	61 (65.6)	154 (82.8)	214 (77.0)
Maternal Occupation			
Employed	17 (18.3)	63 (33.9)	80 (28.7)
Merchant	5 (5.4)	24 (12.9)	29 (10.4)
Housewife	65 (69.9)	91 (48.9)	156 (55.9)
Others	6 (6.5)	8 (4.3)	14 (5.0)
Wealth index			
Lower	58 (62.4)	101 (54.3)	159 (57.0)
Middle	16 (17.2)	49 (26.3)	65 (23.3)
Upper	19 (20.4)	36 (19.4)	55 (19.7)

### Nutritional, anthropometric characteristics and substance abuse

The mean ± SD of maternal height for cases and controls was 155 ± 0.1 cm and 159 ± 0.1 cm, respectively. The percentage of mothers having height of < 150 cm was twice as high among cases compared to controls (23.7% vs 12.4%). Undernutrition in mothers as defined by MUAC < 23 cm was 52.7% and 13.4% among cases and controls, respectively. Mothers who did not receive iron and folate supplementation during pregnancy were 31.2% and 10.8% among cases and controls, respectively. The largest proportion of mothers, 93.5% among cases and 62.9% among controls, had inadequate MDD-W. Nutritional counselling and extra meal consumption during pregnancy were less practiced in mothers from cases. Maternal anemia among cases was 32.3% while it was 9.1% among controls. Maternal khat chewing habit during pregnancy was 35.5% among cases compared to 26.9% among controls. Likewise, mothers who had history of alcohol consumption during pregnancy were 5.4% and 8.1% among cases and controls, respectively (Table 2).

**Table 2:** Distribution of nutritional characteristics among mothers of cases and controls in public health facilities of Dessie Town, Northeast Ethiopia, 2017

Variables	Cases	Controls	Total
	N (%)	N (%)	N (%)
Height (cm)			
< 150	22 (23.7)	23 (12.4)	45 (16.1)
> =150	71 (76.3)	163 (87.6)	234 (83.9)
MUAC (cm)			
< 23	49 (52.7)	25 (13.4)	74 (26.5)
> =23	44 (47.3)	161 (86.6)	205 (73.5)
Iron and folate supplementation			
Yes	64 (68.8)	166 (89.2)	230 (82.4)
No	29 (31.2)	20 (10.8)	49 (17.6)
Any multivitamin use			
Yes	9 (9.7)	36 (19.4)	45 (16.1)
No	84 (90.3)	150 (80.6)	234 (83.9)
Nutritional counseling during pregnancy			
Yes	40 (43.0)	153 (82.3)	193 (69.2)
No	53 (57.0)	33 (17.7)	86 (30.8)
Additional food intake during pregnancy			
Yes	34 (36.6)	134 (72)	168 (60.2)
No	59 (63.4)	52 (28)	111 (39.8)
MDD-W^a^			
inadequate	87 (93.5)	117 (62.9)	204 (73.1)
adequate	6 (6.5)	69 (37.1)	75 (26.9)
Anemia present			
Yes (Hb < 11 g/dl)	30 (32.3)	17 (9.1)	47 (16.8)
No (Hb ≥11 g/dl)	63 (67.7)	169 (90.9)	232 (83.2)
Eating out of home			
Yes	81 (87.1)	167 (89.8)	248 (88.9)
No	12 (12.9)	19 (10.2)	31 (11.1)
Khat chewing			
Yes	33 (35.5)	50 (26.9)	83 (29.7)
No	60 (64.5)	136 (73.1)	196 (70.3)
Alcohol consumption			
Yes	5 (5.4)	15 (8.1)	20 (7.2)
No	88 (94.6)	171 (91.9)	259 (92.8)

^a^MDD-W indicates minimum dietary diversity for women

### Medical and obstetrics characteristics

Proportions of ANC follow up among cases and controls were 89.2% and 96.8%, respectively indicating the majority of mothers in both group had access to maternal care during pregnancy. Mothers who described their pregnancy as unplanned but wanted were 24.7% and 17.2% among cases and controls, respectively. Maternal chronic disease among cases was 14.0% while controls had 15.1%. Maternal pregnancy induced hypertension was 9.7% and 6.5% among cases and controls, respectively. Few mothers among cases and controls, had history of preterm delivery and reactive for HIV test. History of abortion was observed among 23.7% of mothers of cases and 11.8% of mothers of controls. Majority of mothers both in cases and controls were primi-gravida (Table 3).

**Table 3:** Distribution of medical and obstetrics characteristics among mothers of cases and controls in public health facilities of Dessie Town, Northeast Ethiopia, 2017

Variables	Cases	Controls	Total
	N (%)	N (%)	N (%)
Parity			
Primigravida	50 (53.8)	100 (53.8)	150 (53.8)
Multipara	43 (46.2)	86 (46.2)	129 (46.2)
History of abortion			
Yes	22 (23.7)	22 (11.8)	44 (15.8)
No	71 (76.3)	164 (88.2)	235 (84.2)
History of preterm delivery			
Yes	10 (10.8)	5 (2.7)	15 (5.4)
No	83 (89.2)	181 (97.3)	264 (94.6)
ANC follow up			
Yes	83 (89.2)	180 (96.8)	263 (94.3)
No	10 (10.8)	6 (3.2)	16 (5.7)
HIV status			
Reactive	5 (5.4)	4 (2.2)	9 (3.2)
Non- reactive	88 (94.6)	182 (97.8)	270 (96.8)
Pregnancy induced hypertension (PIH)			
Yes	9 (9.7)	12 (6.5)	21 (7.5)
No	84 (90.3)	174 (93.5)	258 (92.5)
Type of pregnancy			
Planned and wanted	66 (71.0)	149 (80.1)	215 (77.1)
Unplanned but wanted	23 (24.7)	32 (17.2)	55 (19.7)
Unplanned and unwanted	4 (4.3)	5 (2.7)	9 (3.2)

### Determinants of low birth weight

In bivariate analyses performed to identify candidate variables for multivariable analysis, variables having *p* < 0.25 were considered candidates for the final multivariable logistic regression model in investigating significant determinants of LBW among study subjects (Table 4).

**Table 4:** Bivariate analyses to identify candidate variables for multivariable logistic regression to identify determinants of LBW, Dessie Town, Ethiopia, 2017

Variables	Cases	Controls	Crude OR (95%CI)	*p*-value
	No %	No %		
Maternal age (year)				
≤ 20	9 (9.7)	32 (17.2)	0.54 (0.25,1.19)	0.127*
21–35	77 (82.8)	147 (79.6)	1	
> 35	7 (7.5)	6 (3.2)	2.24 (0.73,6.91)	0.159*
Residence				
Urban	52 (55.9)	111 (59.7)	1	
Rural	41 (44.1)	75 (40.3)	1.17 (0.71,1.93)	0.548
Educational status of mothers				
Informal	32 (34.4)	32 (17.2)	2.53 (1.42,4.48)	0.002*
Formal	61 (65.6)	154 (82.8)	1	
Occupation of mothers				
Employed	17 (18.3)	63 (33.9)	1	
Merchant	5 (5.4)	24 (12.9)	0.77 (0.26,2.33)	0.646
Housewife	65 (69.9)	91 (48.9)	2.65 (1.42,4.94)	0.002*
Others	6 (6.5)	8 (4.3)	2.78 0.85,9.1)	0.091
Wealth index				
Lower	58 (62.4)	101 (54.3)	1.09 (0.57,2.07)	0.797
Middle	16 (17.2)	49 (26.3)	0.62 (0.28,1.37)	0.235*
Upper	19 (20.4)	36 (19.4)	1	
Height (cm)				
< 150	22 (23.7)	23 (12.4)	2.21 (1.15,4.21)	0.017*
> =150	71 (76.3)	163 (87.6)	1	
MUAC				
< 23	49 (52.7)	25 (13.4)	7.17 (3.99,12.88)	< 0.0001^**^
> =23	44 (47.3)	161 (86.6)	1	
Any multivitamin				
Yes	9 (9.7)	36 (19.4)	1	
No	84 (90.3)	150 (80.6)	2.24 (1.03,4.88)	0.042*
Iron and folate supplementation				
Yes	64 (68.8)	166 (89.2)	1	
No	29 (31.2)	20 (10.8)	3.76 (1.99,7.12)	< 0.0001**
Anemia				
Yes	30 (32.3)	17 (9.1)	4.73 (2.44, 9.17)	< 0.0001**
No	63 (67.7)	169 (90.9)	1	
ANC follow up				
Yes	83 (89.2)	180 (96.8)	1	
No	10 (10.8)	6 (3.2)	3.61 (1.27, 10.28)	0.016*
Nutritional counseling				
Yes	40 (43.0)	153 (82.3)	1	
No	53 (57.0)	33 (17.7)	6.14 (3.52,10.72)	< 0.0001**
Additional food intake during pregnancy				
Yes	34 (36.6)	134 (72)	1	
No	59 (63.4)	52 (28)	4.47 (2.63, 7.61)	< 0.0001**
MDD-W				
inadequate	87 (93.5)	117 (62.9)	8.55 (3.55, 20.61)	< 0.0001**
adequate	6 (6.5)	69 (37.1)	1	
Khat chewing				
Yes	33 (35.5)	50 (26.9)	1.51 (0.88, 2.55)	0.14*
No	60 (64.5)	136 (73.1)	1	
History of abortion				
Yes	22 (23.7)	22 (11.8)	2.31 (1.20, 4.44)	0.012*
No	71 (76.3)	164 (88.2)	1	
History of preterm delivery				
Yes	10 (10.8)	5 (2.7)	4.36 (1.45, 13.16)	0.009*
No	83 (89.2)	181 (97.3)	1	
HIV status				
Reactive	5 (5.4)	4 (2.2)	2.59 (0.68, 9.87)	0.164*
Non-reactive	88 (94.6)	182 (97.8)	1	
Type of pregnancy				
Planned and wanted	66 (71.0)	149 (80.1)	1	
Unplanned but wanted	23 (24.7)	32 (17.2)	1.62 (0.88, 2.98)	0.119*
Unplanned and wanted	4 (4.3)	5 (2.7)	1.81 (0.47, 6.94)	0.389
Infant sex				
Male	49 (52.7)	117 (62.9)	1	
Female	44 (47.3)	69 (37.1)	1.52 (0.92, 2.52)	0.102*

**P < 0.0001 *p < 0.25

In multivariable analysis, selected covariates with *p* < 0.25 in bivariate analyses were entered into the multivariable logistic regression model to control for potential confounders in determining the independent determinants of LBW using backward elimination stepwise likelihood ratio method. Accordingly, absence of iron and folate supplementation, receiving no nutritional counseling and consuming no additional meal during the current pregnancy, and maternal undernutrition, maternal anemia and inadequate MDD-W were found to be significant determinants of low birth weight (Table 5).

**Table 5:** Determinants of LBW in multivariable logistic regression analysis for newborns delivered in public health facilities in Dessie Town, Ethiopia, 2017

Variables	Cases	Controls	COR (95%CI)	AOR [95% CI]
	N(%)	N(%)		
Iron and folate supplementation				
Yes	64 (68.8)	166 (89.2)	1	1
No	29 (31.2)	20 (10.8)	3.76 (1.99,7.12)	2.84 (1.15,7.03)
Nutritional counseling				
Yes	40 (43.0)	153 (82.3)	1	1
No	53 (57.0)	33 (17.7)	6.14 (3.52, 10.72)	4.05 (1.95, 8.38)
Additional meal				
Yes	34 (36.6)	134 (72)	1	1
No	59 (63.4)	52 (28)	4.47 (2.63, 7.61)	3.25 (1.64, 6.44)
Nutritional status				
MUAC < 23	49 (52.7)	25 (13.4)	7.17 (3.99,12.88)	5.62 (2.64,11.97)
MUAC ≥23	44 (47.3)	161 (86.6)	1	1
Anemia				
Yes	30 (32.3)	17 (9.1)	4.73 (2.44, 9.17)	3.54 (1.46,8.61)
No	63 (67.7)	169 (90.9)	1	1
MDD-W				
Inadequate	87 (93.5)	117 (62.9)	8.55 (3.55, 20.61)	6.65 (2.31,19.16)
Adequate	6 (6.5)	69 (37.1)	1	1

*P-value< 0.0001 **p < 0.05

The study showed that mothers who did not take iron and folate supplementation were about three times more likely to deliver LBW babies compared to mothers who did take iron and folate supplementation during current pregnancy [AOR = 2.84: CI (1.15,7.03)]. Mothers who did not receive nutrition counseling during current pregnancy were about four times more likely to deliver LBW babies compared to their counterparts [AOR = 4.05: CI (1.95, 8.38)]. Similarly, mothers who did not take additional meal during current pregnancy were three times more likely to have babies with LBW compared to those mothers who had additional meal during pregnancy [AOR = 3.25: CI (1.64, 6.44)]. Likewise, the odds of giving birth to LBW babies among undernourished mothers compared to their well-nourished counterparts were nearly six fold [AOR = 5.62: CI (2.64, 11.97)]. Anemic mothers had odds of nearly four times to give birth to LBW babies compared to non-anemic mothers [AOR = 3.54: CI (1.46, 8.61)]. Furthermore, mothers who had inadequate dietary diversity had about seven times higher odds of giving birth to LBW babies compared to mothers with adequate dietary diversity [AOR = 6.65: CI(2.31, 19.16)].

## Discussion

The present study revealed that mothers who did not receive nutritional counseling during current pregnancy had higher odds for giving birth to LBW babies in comparison to mothers who did receive nutritional counseling. This might be due to the fact that nutritional counseling might improve women’s food intake and hence, their nutritional status and therefore decrease the risk of delivering LBW babies. This finding was consistent with a study conducted in Gondar University Hospital where mothers who did not have ANC follow up had higher odds of giving LBW babies while this might be ascribed to the routine provision of nutritional counseling during ANC visits [36].

The risk of LBW was higher among mothers who did not receive an additional daily meal during current pregnancy as compared to mothers who did receive an additional meal. This finding was consistent with other similar studies done in Ethiopia [36] and Ghana [38]. Mothers who consumed more food during pregnancy period were 88% less likely to give birth to a LBW infants than those who consumed the same as before pregnancy [38]. Controlled trials have shown that improving meal intakes during pregnancy effectively reduces the risk of giving LBW babies [9]. Likewise, intake of iron and folate supplements during pregnancy had significant association with LBW in our study. Mothers who did not take iron and folate supplementation were more likely to deliver LBW babies than mothers who did take iron and folate supplementation during pregnancy. This finding is in agreement with a study done in Bangladesh that showed intake of iron and folate supplements during pregnancy was found to have a protective effect against LBW [39]. These findings were also consistent with studies done in rural Ethiopia [35], Pakistan [40] and a study from developed country [41]. Iron supplementation during pregnancy protects women from becoming anemic and subsequent increased risk of giving LBW babies, because the required amounts may not be supplied from dietary intake alone during this period [40]. A randomized controlled trial that compared standard iron supplementation with multiple micronutrients during pregnancy on birth size found that women in iron supplementation group had babies with higher birth weight [42]. Furthermore, review of controlled trials suggested 41% reduction in the incidence of IUGR when pregnant mothers had folic acid supplementation, suggesting folate added to antenatal iron could independently affect birth weight of newborns [43]. This is further supported by larger cohort study undertaken in China indicating maternal daily intake of 400 μg of folic acid alone significantly reduced the risks of LBW and SGA status of infants [44].

Moreover, our study showed that anemic mothers had higher odds of delivering LBW neonates compared to non-anemic mothers. This finding was consistent with other studies [13, 39, 45]. Maternal anemia during pregnancy was found to increase odds of giving LBW babies in Yemen [13]. In Bangladesh [41] and India [45, 46] as well as in other settings [15, 18, 20] micronutrients deficiencies during pregnancy had been shown to have serious health implications on the developing fetus and hence, birth size [47]. Anemia could impair oxygen delivery to the fetus and thus interfere with normal intrauterine growth which possibly leads to LBW [19].

Inadequate dietary diversity and undernutrition during pregnancy independently and significantly affected LBW in our study. Mothers having inadequate MDD-W had significantly higher odds of giving birth to LBW babies. This finding was consistent with a study done in rural Ethiopia [35]. Similarly, a study from Ghana showed that women dietary diversity score and dietary patterns had protective effect against LBW [38]. However, recent randomized controlled trial in India reported that an intervention that increased consumption of dairy, fruits, and green leafy vegetables before and during pregnancy through a specially formulated snack had no effect on birth weight, but per-protocol and subgroup analyses showed that there was a possible increase in birth weight if the mother was supplemented for greater than or equal to 3 months before conception and was not underweight [48]. Malshani et al. (2017) showed that second trimester maternal carbohydrate intake had a significant impact on both total gestational weight gain and neonatal birth weight [49].

Undernutrition among mothers as measured by maternal MUAC of less than 23 cm significantly increased the maternal odds of having LBW babies in the present study. This is in agreement with study conducted in Eastern Ethiopia [24] and other countries; Yemen [13] and India [44]. The observed findings might be because anthropometric measurements relate to nutritional status. Even though either acute or chronic maternal malnutrition has direct effect on the birth weight of a baby, acute maternal malnutrition has a more pronounced effect [24].

### Strength and limitation of the study

The present study has some strengths including conducting the study in all public health facilities in the study area, and taking new born weight within 1 h of delivery. Further, using case-control study design can be considered as another important strength of the current study. However, our study has limitations such as not including private health facilities. Also, the completed gestational age was taken from verbal response of respondents. There might be recall bias as respondents had to remember their last date of menstruation, provide intake data for dietary diversity, recall the number of ANC visits, and provide the number of IFA tablet taken. In addition, our study was able to determine the adherence of mothers who were taking iron, folate or multivitamin supplements.

## Conclusion

Lack of iron and folate supplementation, absence of nutritional counseling during pregnancy, lack of additional food during pregnancy, maternal undernutrition (MUAC < 23 cm), maternal anemia and inadequate MDD-W were identified as significant determinants of LBW among newborns included in our study. The importance of nutritional counseling, iron and folate supplementation and additional meal intake during pregnancy, and screening pregnant women for risk of malnutrition and proper identification of high risk-mother needs to be strengthened to reduce the incidence of LBW babies in Ethiopia. In addition, behavioral change communications targeting pregnant women in improving women’s dietary diversity needs to be enhanced by health extension workers and health professionals in each health facility working at ANC clinics. Observational cohort studies and randomized controlled trials with larger sample size are required to confirm the observed associations.
